# TIBIOCALCANEAL ARTHRODESIS: A COMPARISON OF ANTERIOR APPROACH AND TRANSFIBULAR APPROACH

**DOI:** 10.1590/1413-785220233105e267148

**Published:** 2023-10-23

**Authors:** ABDULRAHIM DÜNDAR, DENIZ IPEK

**Affiliations:** 1 Hitit University, Erol Olçok Training and Research Hospital, Department of Orthopedics’ and Traumatology, Çorum, Turkey.

**Keywords:** Ankle Joint, Arthrodesis, Arthritis, Articulação do Tornozelo, Artrodésia, Artrite

## Abstract

**Objective:**

The aim of this study was to evaluate the clinical and radiologic results and complications of patients who underwent ankle arthrodesis performed by the transfibular approach and anterior approach in end-stage ankle osteoarthritis.

**Methods:**

Between 2016 and 2022, 41 patients who satisfied the inclusion criteria for this retrospective comparative analysis were included. Of them, 19 patients are included in the anterior approach group and 22 patients are included in the transfibular approach group. The mean age of the participants is 58.9 years. Collected data included the BMI, American Orthopedic Foot and Ankle Society (AOFAS) hindfoot scale, visual analogue scale (VAS) score, diabetes, smoking, time to fusion, nonunion, union rate, preoperative and postoperative coronal tibiotalar angle and complications.

**Result:**

The mean time to bone union was 14.3 weeks (range 11-17 weeks) in the anterior approach group, and 11.3 weeks in the transfibular approach group. Statistically significant difference was found between the two groups. Nonunion occurred in one case in the transfibular approach group and three cases in the anterior approach group. There was no significant difference in the nonunion rate between the both groups (p = 0.321). VAS score, and AOFAS score of the two groups were similar and no significant differences were found (p = 0.491, p = 0.448, p = 0.146, p = 0.073, p = 0.173, p = 0.506, respectively).

**Conclusions:**

A stable and firm ankle arthrodesis and plantigrade foot can be achieved with both transfibular approach and anterior approach technique. ***Level of Evidence III, Retrospective Comparative Study.***

## RESUMO

**Objetivo:**

Avaliar os resultados clínicos e radiológicos e as complicações de pacientes submetidos à artrodese de tornozelo realizada por abordagem transfibular e abordagem anterior em fase terminal de osteoartrite de tornozelo.

**Métodos:**

Foram incluídos 41 pacientes atendidos entre 2016 e 2022 que satisfizeram os critérios de inclusão para esta análise comparativa retrospectiva. Destes, 19 incluídos no grupo de abordagem anterior e 22 no grupo de abordagem transfibular. A média de idade foi de 58,9 anos. Os dados coletados incluíram o índice de massa corporal (IMC), a escala de retropé da American Orthopedic Foot and Ankle Society (AOFAS), o escore da escala visual analógica (EVA), assim como a presença de diabetes, tabagismo, tempo de fusão, não união, taxa de união, ângulo tibiotalar coronal pré e pós-operatório e complicações.

**Resultado:**

O tempo médio de consolidação óssea foi de 14,3 semanas (variação de 11 a 17 semanas) no grupo de abordagem anterior e 11,3 semanas no grupo de abordagem transfibular. Foi encontrada diferença estatisticamente significante entre os dois grupos. A consolidação não ocorreu em um caso no grupo de abordagem transfibular e em três casos no grupo de abordagem anterior. Não houve diferença significativa na taxa de não consolidação entre os dois grupos (p = 0,321). Os escores nas escalas EVA e AOFAS dos dois grupos foram semelhantes, não sendo encontradas diferenças significativas (p = 0,491, p = 0,448, p = 0,146, p = 0,073, p = 0,173, p = 0,506, respectivamente).

**Conclusões:**

Uma artrodese estável e firme do tornozelo e um pé plantígrado podem ser obtidos tanto com a abordagem transfibular quanto com a técnica de abordagem anterior. **Nível de Evidência III, Estudo Comparativo Retrospectivo.**

## INTRODUCTION

### Objective

The most famous and commonly used procedure for end-stage ankle arthritis is still open ankle arthrodesis.^1^ It has been accomplished using a variety of surgical procedures, including plates, intramedullary nails, screws, external fixators, and a combination of these techniques.^2^ ^),(^ ^3^

Tibiotalar joint stabilization and stiff fixation are the goals of ankle arthrodesis. A plantigrade foot is made possible by a stable ankle arthrodesis. Ankle arthrodesis has, however, been linked to several significant complication. Nonunion of the tibiotalar joint is the most significant and frequent complication.

Currently, the transfibular, anterior, medial, and posterior methods, among others, have all been documented for tibiotalar arthrodesis.^4^ Because they offer a larger surgical field of vision, the anterior approach and transfibular approach have become more popular in recent years. However, DeHeer et al.^5^ found that utilizing the transfibular technique, it is challenging to obtain the right tibiotalar deformity on the coronal plane. In one study,^6^ comparable fusion rates for each method were observed, although the outcomes in terms of complications varied. To the best of our knowledge, there is, however, relatively little research that compares anterior and transfibular techniques.

This retrospective study’s objective was to assess the clinical, radiological, and postoperative complications of ankle arthrodesis in patients with ankle arthritis carried out by anterior and transfibular methods.

## METHODS

The research protocol was approved by the Hitit University Ethics Committee (04.11.2022-23), and informed consent was obtained from all patients.

### Study patients

41 patients who received transfibular or anterior tibiotalar arthrodesis between February 2016 and February 2022 and were monitored for more than a year were included in this study. Patients with septic arthritis, neuropathic arthritis, and follow-up periods of less than two months were excluded. Before surgery, all patients with ankle arthritis had conservative care. If persistent discomfort around the ankle made movement impossible, however, they undergo either fusion of the ankle using an anterior or transfibular method. The study comprised a total of 41 patients.

Demographic information, BMI, the AOFAS hindfoot scale, the VAS score, diabetes, smoking, the time to fusion, the nonunion rate, the union rate, the preoperative and postoperative coronal tibiotalar angle, and complications were all noted. The AOFAS hindfoot scale and VAS score were used to evaluate the clinical assessment.

### Radiological assessment

To validate the union of the ankles joint and the position of the tibiotalar joint, anterior-posterior, and lateral X-ray views were examined. The presence of trabecular lines at the point of contact and the removal of the radiolucent line between the talus and tibia on the anterior-posterior and lateral X-ray views were used to verify the bony union monthly.^6^ The coronal tibiotalar angle was assessed for postoperative varus/valgus deformity using the angle between the long axis of the tibia and the calcaneus on the hindfoot anterior-posterior X-ray.^7^

### Surgical techniques

#### Transfibular approach

**Figure 1:**
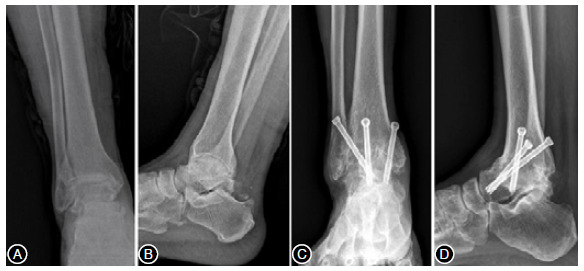
Preoperative weightbearing radiographs of the ankle. Takakura stage 4. (A) anteroposterior X-ray and (B) lateral X-ray. Anterior approach was performed with three 6.5-mm cannulated screws. Ankle anteroposterior (C) and lateral (D) radiographs view showing fusion of the ankle joint at the final follow up (13 months after operation).

An approximately 6 to 8 cm lateral single incision was made across the distal fibula while the patient was in the lateral decubitus posture and under spinal or general anesthesia. The fibula was cut with Oscillating Saw at approximately 5 cm proximal to the ankle joint, and the osteotomized fragment was transected in the sagittal plane for use as a bone transplant. The articular cartilage of the ankle joint was observed using a lamina spreader. Osteophyte and arthritic articular cartilage on the tibia and talus were both removed. Following joint preparation, the arthrodesis was carried out using three 6.5-mm cancellous screws that are partially threaded to accomplish the ankle alignment in 0°-5° abduction, 5°-10° external rotation, and neutral plantar flexion. Two of them were positioned from the anterolateral side of the tibia to the medial side of the talus, and one 6.5-mm cancellous lag screw was positioned across the talus from the anteromedial to the posteroinferior. Two parallel 4.5 mm cortical screws were used to secure the onlay distal fibular graft (Figure 1).

#### Anterior approach

**Figure 2:**
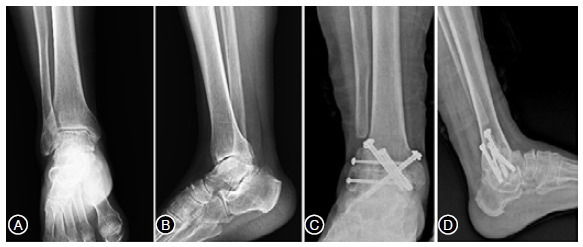
Preoperative weightbearing radiographs of the ankle. Takakura stage 3. (A) anteroposterior X-ray and (B) lateral X-ray. Transfibular approach was performed with three 6.5-mm cannulated screws and two 4.5 cannulated screws with fibular onlay bone graft; (C) anteroposterior view; (D) lateral view showing fusion of the ankle joint at the final follow up (11 months after operation).

An 8-10 cm long longitudinal single incision was made over the anterior tibial tendon while the patient was supine and under spinal or general anesthesia. The articular cartilage of the tibial plafond, the talar dome, and the osteophyte were removed, together with the anterior joint capsule. Under fluoroscopic guidance, three partly threaded 6.5-mm cancellous screws were used to fix the arthrodesis after joint preparation. One 6.5-mm screw was inserted from the anterolateral side of the tibia to the medial side of the talus and two 6.5-mm cannulated screws were inserted into the anteromedial side of the tibia from the lateral side of the talus (Figure 2).

### Postoperative management

On the 14th postoperative day, the sutures were removed in both surgical techniques. Following surgery, a short leg cast was applied for two to four weeks. A walking boot was used for an additional four weeks after the cast was removed. Patients were restricted from bearing any weight for the first month following surgery. After that, they were permitted to bear some weight, and after radiographic evidence of bone union, they were permitted to full weight-bearing ambulation. Serial radiographs were taken at 2, 4, 6, and 12 months as well as every year after that until the bone had fused. On follow-up radiographs, the coronal tibiotalar angle, implant location, and bony union were all assessed. The postoperative rehabilitation process was the similar in both groups.

### Statistical analysis

Statistical analyses of the data were performed with the SPSS (Version 22, SPSS Inc., Chicago, IL, USA, Undergraduate: Hitit University) software. Descriptive statistics were reported using numbers and percentages for categorical variables, and mean ± standard deviation or median (minimum-maximum) depending on data distribution for numerical variables. The normal distribution test of numerical data was evaluated with the Shapiro Wilks test. Mann Whitney U test was used in the comparison of numerical data between two independent groups, since parametric test assumptions were not provided. Chi-square or Fisher’s exact test was used depending on the sample sizes in the crosstab cells for ratio comparisons between research groups. p < 0.05 was accepted for statistical significance level in all comparisons.

## RESULT

Between 2016 and 2022, 41 patients who satisfied the inclusion criteria for this retrospective comparative analysis were included. Of them, 19 patients are included in the group AA (anterior approach) and 22 patients are included in the TF (transfibular) group. The mean age of the participants is 58.9 ± 9.13 years. The study included 12 (% 29.3) women and 29 (%70.7) men. Body mass index (BMI) was 26.79 2.71 kg/m^2^ on average. Regarding sociodemographic and clinical statistics, the results of the study showed that there is no statistical difference between two groups (Table 1).

**Table 1:** Comparison of socio-demographic and clinical data of patients between research groups.

		Anterior approach (n = 19)	Transfibular approach (n = 22)	p values
Gender	Male	13 (68.4%)	16 (72.7%)	0.763^a^
	Female	6 (31.6%)	6 (27.3%)	
Diabetes mellitus	No	4 (21.1%)	5 (22.7%)	1.000^b^
	Yes	15 (78.9%)	17 (77.3%)	
Smoking	No	5 (26.3%)	7 (31.8%)	0.699^a^
	Yes	14 (73.7%)	15 (68.2%)	
Complication	No	16 (84.2%)	22 (100%)	0.091^b^
	Yes	3 (15.8%)	0	
Diagnose	Trauma	13 (68.4%)	14 (63.6%)	0.747^a^
	Primer artrit	6 (31.6%)	8 (36.4%)	
Age		57.16 ± 8.44	60.41 ± 9.62	0.261^c^
BMI (kg/m^2^)		25.62 ± 2.44	26.8 ± 2.57	0.329^c^
**VAS score**				
Preoperative		7 (4-8)	7 (4-8)	0.491^d^
		(6.32 ± 1.33)	(6.59 ± 1.29)	
Postoperative		2 (0-4)	2 (0-4)	0.448^d^
		(2.37 ± 1.11)	(2.09 ± 1.26)	
**AOFAS score**				
Preoperative		43 (27-56)	47.5 (31-56)	0.146^d^
		(42 ± 8.28)	(45.5 ± 6.33)	
Postoperative		78 (58-86)	81 (67-89)	0.073^d^
		(75.5 ± 7.88)	(79.2 ± 6.36)	
**Coronal angle**				
Preoperative		78 (54-102)	75.5 (54-102)	0.173^d^
		(80.7 ± 11.8)	(75.2 ± 13.6)	
Postoperative		87 (83-90)	88 (84-90)	0.506^d^
		(86.7 ± 2.01)	(87.2 ± 1.87)	

a Chi square test with n (%); ^b^ Fisher exact test with n (%); ^c^ Student’s t-test with mean ± standard deviation; ^d^ Mann Whitney U test with median (min-max) and mean ± standard deviation.

Among the research groups, the distribution of gender, diabetes, smoking conditions, complication incidence rates, and diagnosis status were statistically similar (p = 0.763, p = 1.000, p = 0.699, p = 0.091, p = 0.747) respectively with each other. The average age and the mean BMI of the two groups has no statistically significant difference (p = 0.261 and p = 0.329) respectively. Preoperative and postoperative coronal tibiotalar angle, VAS score, and AOFAS score of the two groups were similar and no significant differences were found (p = 0.491, p = 0.448, p = 0.146, p = 0.073, p = 0.173, p = 0.506, respectively Table 1). In the AA group, the mean VAS and AOFAS ankle-hindfoot functional score improved from 6.32 (range, 4 to 8) and 43 (range, 27 to 56) preoperatively to 2.37 (range, 0 to 4) and 78 (range, 58 to 86) at the final follow-up, respectively. In the TF group, the mean VAS and AOFAS ankle-hindfoot functional score improved from 7 (range, 4 to 8) and 47.5 (range, 31 to 56) preoperatively to 2 (range, 0 to 4) and 81 (range, 67 to 89) at the final follow-up, respectively.

There was no noteworthy difference in the nonunion rate between the both groups (p = 0.321). The mean time to bone union in the AA group was 14.3 weeks (range 11-17 weeks), while it took 11.3 weeks in the TF group (range 9-13 weeks), statistically significant difference was found between the two groups (p < 0.001) (Table 2). The distribution of the time to fusion values between the two groups was shown in Boxplot and Figure 1.

**Table 2:** Comparison of non-union rates and time to fusion values of patients between research groups.

		Anterior approach (n = 19)	Transfibular approach (n = 22)	p values
Non-union	No	16 (84.2%)	21 (95.5%)	0.321^a^
	Yes	3 (15.8%)	1 (4.5%)	
Time fusion		14 (11-17)	11.5 (9-13)	< 0.001^b^
		(14.3 ± 1.48)	(11.3 ± 1)	

a Fisher exact test with n (%); ^b^ Mann Whitney U test with median (min-max) and mean ± standard deviation.

## DISCUSSION

Open ankle arthrodesis is the most common surgical procedure for end-stage ankle osteoarthritis.^8^ The literature has reported several surgical techniques, including the transfibular, anterior, and posterior approaches.^4^ However, transfibular technique or anterior approach technique have been used for the most of the open ankle arthrodesis procedures.^9^ The ankle joint surface can be seen in great detail from the anterior approach. This method, however, can harm the front vasculature and has limited exposure to the posterior malleoli.^5^ The anterior technique, which similarly has limited benefit in instances with significant varus-valgus deformity or bone loss, is more likely to cause damage to the superficial peroneal nerve and the neurovascular system anterior tibia.^10^

There is less danger of complications such as wound dehiscence, infection, and prolonged recovery in the transfibular method than in the anterior route because the delicate tissues are less denser on the anterior side than on the lateral side. The subtalar joint, sinus tarsi, and ankle joint are all exposed during the transfibular process, which is useful for deformity treatment. It permits extensive contact areas, great stability, and fixation utilizing a fibular strut graft in addition to simple rectification of abnormalities such as ankylosis.

The transfibular approach is a widely utilized technique today due to the superb lateral joint visibility and simple repair of sagittal abnormalities.^11^ The transfibular method does have some drawbacks, though, including challenges with plate attachment, coronal plane correction, and medial gutter debridement.^12^

According to Nielsen, Linde, and Jensen,^13^ between 77% and 100% of patients who underwent open surgery with internal fixation experienced fusion. The anterior technique has a 100% success rate, according to Gordon et al.^14^ The transfibular method was recommended by Holt et al.,^15^ who also noted fusion rates of 93%. The rates of union were 95.4 % and 89.5 %, respectively, according to Kim et al.’s^11^ in the comparison of the groups i.e. anterior approach group with the transfibular approach group. 41 patients with advanced ankle arthritis were examined in our study. Regarding the incidence of nonunion and hindfoot alignment, between the two groups, there was no substantial distinction statistically. In the anterior approach group, there were three nonunion instances and one in the transfibular approach group. In the transfibular group, we attained a union rate of 95.5 percent, while in the anterior approach group, we achieve a union rate of 84.2 percent. Regarding clinical outcomes, union rate, and postoperative complications, our results were comparable to those of earlier research.^16^ ^),(^ ^17^

### Limitation of the study

This study has some limitations, including a retrospective methodology and a limited patient population. Therefore, additional prospective randomized studies comparing the anterior approach and the transfibular technique with more patients are needed to confirm the best course of action for end-stage ankle arthritis.

## CONCLUSION

To conclude, we found that the transfibular technique and anterior technique were effective in managing end-stage ankle arthritis. A stable and firm ankle arthrodesis and plantigrade foot can be achieved with any surgical technique. However, the transfibular method allows for quicker mobilization and recuperation and adds additional support with the fibular onlay graft.
